# The Role of Syncytin in Placental Angiogenesis and Fetal Growth

**DOI:** 10.3389/fcell.2022.852561

**Published:** 2022-04-12

**Authors:** Ya-Nan Wang, Yixin Ye, Da Zhou, Ze-Wen Guo, Zhelei Xiong, Xing-Xing Gong, Shi-Wen Jiang, Haibin Chen

**Affiliations:** ^1^ Department of Histology and Embryology, Shantou University Medical College, Shantou, China; ^2^ Department of Obstetrics and Gynecology, The Second Affiliated Hospital of Shantou University Medical College, Shantou, China; ^3^ Department of Obstetrics and Gynecology, Shantou Central Hospital, Shantou, China; ^4^ Center of Reproductive Medicine, The Affiliated Wuxi Maternity and Child Health Care Hospital of Nanjing Medical University, Wuxi, China

**Keywords:** syncytin, placental angiogenesis, syncytiotrophoblast, pathological placenta, PI3K/Akt/mTOR pathway

## Abstract

**Background:** Syncytin, a retroviral envelope protein, is specifically expressed on trophoblast cells and mediates formation of the syncytiotrophoblast through fusogenic activity. Decreased expression of Syncytin was found in fetal growth restriction placentas.

**Results:** By generating an inducible knockout of the *syncytin-a* gene in mice, we show a specific disruption of placental angiogenesis with abnormal formation of two syncytiotrophoblast layers. Consistent with the defects observed *in vivo*, conditioned medium collected from trophoblast cells, following Syncytin-1 knockdown, contains lower expression of vascular endothelial growth factor and placental growth factor, and higher levels of soluble fms-like protein kinase-1 in BeWo and HTR-8/SVneo cells which related with suppressed PI3K/Akt/mTOR pathway, and is reduced in ability to induce tube formation by HUVECs.

**Conclusion:** Syncytin participates in angiogenesis during placental development was first identified both *in vivo* and *in vitro*. Here, we give a new sight on understanding syncytin and pathophysiology of placenta related disease such as fetal growth restriction.

## Introduction

Placental angiogenesis is critical to the success of pregnancy due to its importance in providing the optimum exchange environment, for oxygen, nutrient and waste, to support fetal development (Sherer and Abulafia, 2001; Mihu et al., 2009). Normal development of fetus and placentas all depend on successful establishment of early placental angiogenesis. A lot of proangiogenic and anti-angiogenic molecules are involved in this complex process of placental angiogenesis (Demir et al., 2007; Pandya et al., 2016; Melincovici et al., 2018; Basak et al., 2020; Sandovici et al., 2022). Well-regulated angiogenesis plays a crucial role in placental and fetal development (Pratt et al., 2015), and defective placental angiogenesis is associated with several pregnancy complications, such as preeclampsia (PE), fetal growth restriction (FGR) and abortion (Furuya et al., 2011; Borras et al., 2014; Ozturk et al., 2017; Helmo et al., 2018).

As an endogenous retroviral envelop protein, syncytin is specifically expressed in placental trophoblast cells and plays an essential role in placental syncytiotrophoblast formation through fusogenic activity (Gong et al., 2005). Two pairs of endogenous retroviral *env* genes of humans (*HERV-W* and *HERV-FRD*) and mice (*syncytin-a* and *syncytin-b*) were identified to encode syncytin proteins Syncytin-1/Syncytin-2 and Syncytin-A/Syncytin-B, respectively (Blaise et al., 2005; Dupressoir et al., 2005; Esnault et al., 2008; Noorali et al., 2009; Dupressoir et al., 2011). Labyrinth begins formation with expression of *GCM-1* and *syncytin* genes at E8.5 d and well formation of placental structures can be observed at E10 d (Anson-Cartwright et al., 2000; Yu et al., 2002; Shi et al., 2004). High expression levels of *syncytin-a* and *syncytin-b* genes kept from E11.5 to E17.5 d (Dupressoir et al., 2005; Asp et al., 2007). In human and murine placentas, cytotrophoblast cells continuously fuse with the overlying syncytiotrophoblast layers through fusogenic process by syncytin to sustain barrier function and transport activities (Pötgens et al., 2002). Moreover, the non-fusogenic functions of syncytin leads to apoptosis and proliferation, also has been found to be involved in pathological placenta formation (Huang et al., 2013; Huang et al., 2014).

Syncytin-1 and syncytin-2 were decreased expressed in FGR placentas compared to the control patients (Langbein et al., 2008; Ruebner et al., 2010; Ruebner et al., 2013). Growing evidence showed altered placental vascular anatomy and impaired vascular function in FGR placentas, alongside endothelial dysfunction and hypoxia, which indicating a potential mechanism in abnormal placental pathology in FGR placentas (Maulik et al., 2006; Tun et al., 2019; Boss et al., 2021; Liu et al., 2021; Morley et al., 2021). Angiogenesis and proangiogenic factors, such as vascular endothelial growth factor (VEGF) and placental growth factor (PLGF) are essential mediators in placental angiogenesis and play a crucial role in normal placental development (Demir et al., 2007; Chen and Zheng, 2014). In FGR patients, antiangiogenic receptors, such as the soluble fms-like protein kinase 1 (sVEGFR, sFlt-1) and soluble endoglin (sEng), are detectable in the circulation and placentas (Vogtmann et al., 2019; Pei et al., 2021). These antiangiogenic receptors suppress VEGF- and PLGF-mediated angiogenic signaling, resulting in dysfunction of endothelial cells (Helmo et al., 2018; Zhang et al., 2021). Interruption of Syncytin-A resulted in widespread vascular abnormalities in placental labyrinth layer, indicating that Syncytin-A disruption maybe involved in poor placental angiogenesis (Dupressoir et al., 2009; Qiao et al., 2017).

Here, we report a new non-fusogenic function whereby syncytin mediates placental angiogenesis. The placentas of *syncytin-a* knockout mice exhibit deficiencies in syncytiotrophoblast formation and fetal blood vessel development with low expression of VEGF and PLGF of placenta tissues. Consistent with the *in vivo* findings, the *HERV-W*-silenced trophoblast cells (BeWo cells and HTR-8/SVneo cells) showed low expression levels of VEGF and PLGF, and led to a poor tube formation upon co-culturing with HUVECs. The discovery of syncytin as a critical protein in angiogenesis and placental development may provide new insight on the pathological analysis of FGR placenta.

## Materials and Methods

### Mice and Genotyping

The *CreERT2/SynA* ^*fl/fl*^ mouse used in our study has been previously described (Qiao et al., 2017). Mice were housed in an SPF environment at the Animal Central of Shantou University Medical College. All procedures were performed with the approval of the Animal Experimental Ethical Inspection (SUMC2020-308). Both *Cre* and *SynA* ^*fl/fl*^ genes were genotyped by PCR. Primers for these genes are listed in Table 1. The expected product for *Cre* and *SynA* ^*fl/fl*^ gene were 530 bp and 367 bp in size, respectively. Female and male mice of *CreER* ^*T2*^ */SynA* ^*fl/fl*^ genotype were mated at a male∶female ratio of 1∶2 in the same cage at 4:00～8:00 p.m., and were separated at 8:00～9:00 a.m. the next day. Meanwhile, embryonic day (E0.5 d) was recorded when a vaginal plug was checked. Tamoxifen (Sigma) was dissolved in the sunflower seed oil at 10 mg/ml, and intraperitoneal injection of this mixture (0.05 mg/g) was administered at E11.5 d to induce the *syncytin-a* knockout genotype of *CreER* ^*T2*^ */SynA* ^*−/−*^ placenta. Pure sunflower seed oil was injected into mouse with the same dose at E11.5 d as a control. Mice were sacrificed from E14.5 to E18.5 d after injection, embryos and placentas were harvested immediately.

**TABLE 1:** Sequences of primers for genotyping.

Gene	Sequence
*Cre-F*	CCACGACCGTGACAGCAA
*Cre-R*	TGA​CCA​GAG​TCA​TCC​TTA​GCG
*SynA* ^*fl/fl*^ *-F*	CTA​CTA​ACC​AGA​GGC​ATT​GAA​GCT​C
*SynA* ^*fl/fl*^ *-R*	GAT​GCA​GGA​GAA​TCT​CTG​TGA​GTT​C

### Morphological Analysis

Freshly collected embryos and placentas from E14.5 to E18.5 d were photographed with a stereoscope. Paraffin samples of embryos and placentas were made following fixation in 4% paraformaldehyde buffer. Then paraffin samples were sectioned at 5 μm with a Leica RM2235 microtome for histological staining. H&E staining of embryos was performed by using a hematoxylin and eosin kit (Baso). Immunofluorescence staining of placenta was performed with anti-Monocarboxylate Transporter 1 (anti-MCT1) antibody and anti-Monocarboxylate Transporter 4 (anti-MCT4) antibody. Anti-MCT1 antibody was used to label Syncytiotrophoblast-I (STB-I) and anti-MCT4 antibody was used to label Syncytiotrophoblast-I (STB-II). Fetal vessels were stained with anti-CD34 antibody, and MVD was calculated according to the method of Weidner (40×) where single endothelial cells or clusters labeled with CD34 in brown-yellow color, regardless of whether a lumen was formed or whether there were red blood cells in the lumen, were counted (Weidner, 1995). However, if the lumen diameter was greater than eight red blood cells or there was obvious muscularization surrounding the endothelial cells, they were not counted. Alkaline phosphatase (AP) staining was used to label sinusoidal trophoblast giant cells (Stgcs) for assessing the maternal lacunas.

### Placental Transmission Electron Microscopy

Placenta tissues were fixed in 2.5% glutaraldehyde, dehydrated through a serious of 50%–100% ethanol, and then incubated in a 1:9 mixture of A mixture (Epon812: DDSA = 31 : 50) and B mixture (Epon 812: NMA = 100: 89), and stirred while DMP-30 was slowly added. Ultrathin sections (1 µm) were stained with uranyl acetate and lead citrate. Experiments were performed in the electron microscopy lab of Shantou University Medical College. Sections were observed with a JEM-F200 transmission electron microscope (JEOL, Japan).

### TUNEL Staining

TUNEL staining of placental tissues was performed by using *In situ* Cell Death Detection Kit (Roche, 11684817910). Anti-MCT1 and Anti-MCT4 antibodies were co-stained to label STBs. Negative control was set in every experiment.

### Cell Culture and Transfection

BeWo cells were purchased from the Wuhan Cell Banker (Guangdong Peiyu) and cultured in Ham’s F-12K medium (Pythonbio) supplemented with 15% fetal bovine serum (Gibco) and 1% penicillin/streptomycin (Gibco). HTR-8/SVneo cells were a gift from Prof. Xue-Song Yang (Jinan University) and cultured in DMEM medium (Sigma) supplemented with 10% fetal bovine serum (Gibco) and 1% penicillin/streptomycin (Gibco). Human umbilical vein endothelial cells (HUVECs) were a gift from Prof. Xiao-Jun Yang (Shantou University Medical College) and cultured in DMEM medium (Sigma) supplemented with 15% fetal bovine serum (Gibco) and 1% penicillin/streptomycin (Gibco). Transfection of *HERV-W* siRNA (GenePharma) was performed using Lipofectamine 2000, the sequences were 5′-GGC​GGU​AUC​ACA​ACC​UCU​ATT-3′ and 5′-UAG​AGG​UUG​UGA​UAC​CGC​CAA-3’. Forty-8 h after transfection, transfection efficiency was detected by using real-time quantitative PCR. All siRNA experiments were conducted in both BeWo and HTR-8/SVneo trophoblast cells, and data showed in this work was from BeWo cells.

### Real-Time Quantitative PCR

Total RNA of placental tissues and trophoblast cells were extracted with Trizol (Invitrogen). The concentration of RNA was determined by a Nanodrop 2000 (Thermo) and 1 μg RNA was reversed-transcribed into cDNA using HiScript® III RT SuperMix (Vazyme). Real-time quantitative PCR were performed on the SLAN-96S PCR system (Shanghai Hongshi). Primer sequences are listed in Table 2.

**TABLE 2:** Sequences of primers for real-time quantitative PCR.

Gene	Sequence
*GAPDH-F*	GTT​ACC​AGG​GCT​GCC​TTC​TC
*GAPDH-R*	GAT​GGT​GAT​GGG​TTT​CCC​GT
*syncytin-a-F*	AAT​GGA​GAA​ACC​CCT​TAC​GCT
*syncytin-a-R*	GTG​GGG​TAG​GTG​TGG​CAG​TG
*VEGF-F*	TCA​TCA​GCC​AGG​GAG​TCT​GT
*VEGF-R*	GGG​AGT​GAA​GGA​GCA​ACC​TC
*PLGF-F*	GAG​GAA​CCC​CAC​CTG​TGA​TG
*PLGF-R*	ATT​CAG​CAG​GGA​CGA​GTT​GG
*sFlt-1-F*	CGC​CAC​CTC​CTG​CTT​CAA​AAC
*sFlt-1-R*	TAC​GCT​GAG​CTT​TCC​ACG​CA

### Immunoblotting

Trophoblast cells were lysed with RIPA mixed buffer (RIPA: PMSF: protease inhibitor cocktail = 98:1:1), then thoroughly disrupted by ultrasonication. Protein concentration was determined using the BCA Protein Assay Kit (Beyotime, P0012S). Proteins were separated by 10% SDS-PAGE (EpiZyme, PG112), blotted onto a polyvinylidene difluoride membrane and incubated with anti-PI3K antibody (Proteintech, 60225-1-Ig), anti-Akt antibody (Proteintech, 10176-2-AP), anti-p-Akt antibody (Cell Signaling, #4060) and anti-mTOR antibody (Proteintech, 60888-1-Ig) with gentle shaking overnight at 4°C. Following three TBST washes, the membrane was then incubated with HRP-conjugated secondary antibodies for 2 h at room temperature. Bands were visualized using enhanced chemiluminescence reagents (Thermo, 34580).

## ELISA

The expression levels of VEGF, PLGF and sFlt-1 in *HERV-W*-silenced trophoblast cells and cell culture supernatant were detected by using the ELISA kits (CUSABIO).

### *In vitro* Tube Formation Assay

HUVECs were seeded on 96-well plates and co-cultured with conditional medium collected from control and *HERV-W-*silenced trophoblast cells. Plates were pre-coated with Matrigel (Corning) and placed in a 37°C incubator for 1 h to allow solidification. Then, HUVECs were planted on the Matrigel after pre-treated with conditioned medium for 2 h and observed by using inverted microscope (Figure 4A). Tube formation was analyzed by ImageJ software.

### Statistical Analysis

At least three independent replicates were performed for all experiments, and the data obtained are expressed as mean ± SD. Pregnant mice were performed at E14.5, E15.5, E16.5, E17.5 and E18.5 d, and the total number of placentas and littermates obtained was 480 (E14.5 d, *n* = 52; E15.5 d, *n* = 65; E16.5 d, *n* = 100; E17.5 d, *n* = 182; E18.5 d, *n* = 81). Statistical analysis was performed using SPSS software. Student’s two-tailed *t* test and one-way ANOVA were used to identify significant differences. For small samples, the independent-samples *t* test of none-parametric methods was used to identify significant differences.

## Results

### Morphological Defects of *CreER* ^*T2*^ */SynA* ^*−/−*^ Embryos and Placentas

**FIGURE 1:**
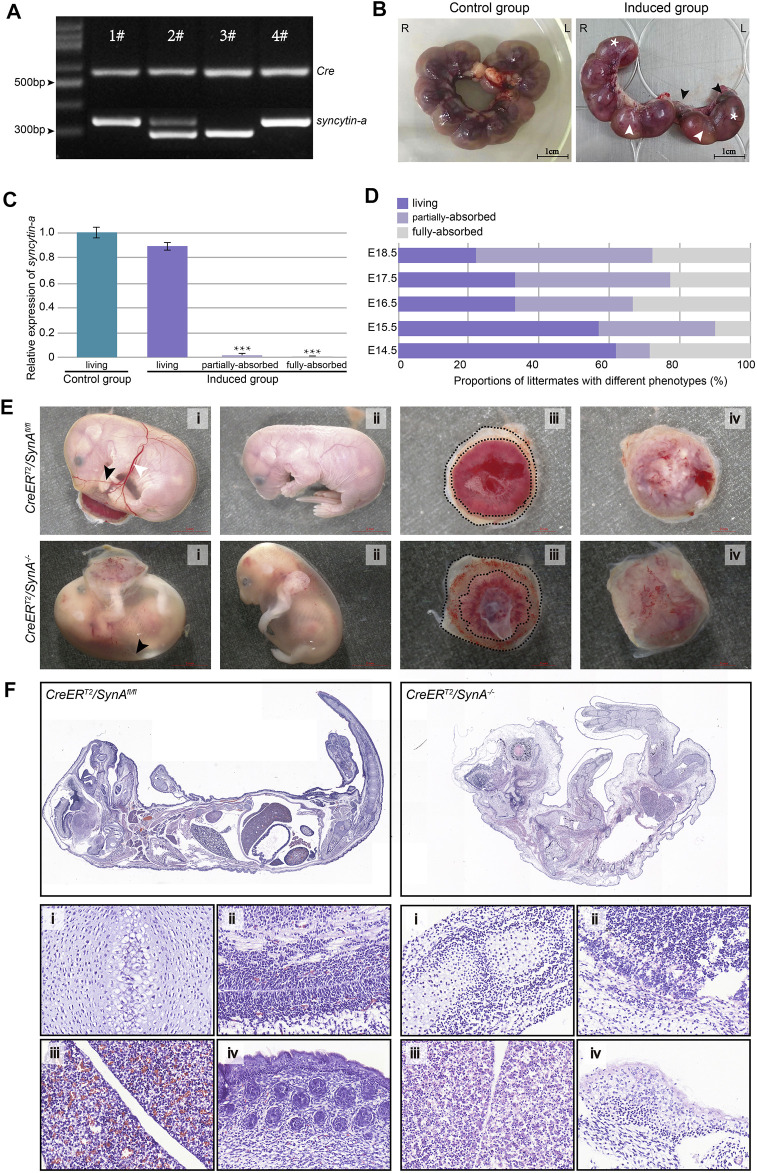
*CreER* ^*T2*^ */SynA* ^*−/−*^ mouse and the phenotype of placentas and fetuses. **(A)** Genotyping of newborn mouse pups for the *CreER* ^*T2*^ */SynA* ^*fl/fl*^ genotype (1# & 4#). The expected products for *Cre* and *SynA* ^*fl/fl*^ gene were 530 bp and 367 bp in size, respectively. **(B)** The tamoxifen-induced group showed markedly abnormal intrauterine littermates (E17.5 d). Intrauterine littermates were arranged regularly and evenly in the uninduced group but composed of living (*) and partially/fully-absorbed (black/white arrow) littermates in the induced group. **(C)** Relative expression of *syncytin-a* gene in placentas from living, partially-absorbed and fully-absorbed embryo in the induced group compared to living embryo in the uninduced group. Placentas with partially-absorbed embryos and fully-absorbed embryos were determined as *CreER* ^*T2*^ */SynA* ^*−/−*^(E17.5 d). ***: *p* < 0.001. **(D)** Proportion of littermates with different phenotypes in the induced group. The proportion of absorbed littermates increased gradually as gestation progressed from E14.5 to E18.5 d. **(E)** Representative embryos and placentas of *CreER* ^*T2*^ */SynA* ^*−/−*^ and *CreER* ^*T2*^ */SynA* ^*fl/fl*^ genotypes (E18.5 d). **(F)** H&E staining of representative embryos (E18.5 d). (i) Bone, (ii) brain, (iii) liver and (iv) skin.

*CreER* ^*T2*^ */SynA* ^*fl/fl*^ mice were generated for inducible disruption of *syncytin-a* gene (Figure 1A, -#1, #4). After injection of tamoxifen at E11.5 d, the embryos and placentas were collected from E14.5 to E18.5 d. Tamoxifen was replaced with pure sunflower oil in the uninduced group. Noteworthily, the embryos and placentas were abnormal in the induced group, with embryos either alive, partially absorbed or fully absorbed (Figure 1B). Placentas connected with the latter two were *syncytin-a* ^*−/−*^, while *syncytin-a* gene was still highly expressed in the placentas of the surviving embryos, as determined by genotyping (Figure 1C). The proportion of absorbed littermates increased gradually as gestation progressed from E14.5 to E18.5 d (Figure 1D). Clearly, the absorbed embryos were smaller and paler. Decreased vascularization was observed at the surface of the yolk sac with fewer branches often presented as indistinct white vessels that were difficult to recognize. Moreover, the placentas were paler with more extra-amniotic tissue (Figure 1E). To further analyze the embryonic defects, we performed histological analysis of living embryos from an uninduced mice and absorbed embryos from induced mice. The overall shape difference between them was significant. Organs from control living embryos of uninduced group appeared normal but were hard distinguish in the absorbed embryos and were infiltrated with inflammatory cells (Figure 1F). Previous studies also identified a low fetus body weight (Qiao et al., 2017). These defects were displayed from E14.5 to E18.5 d and may have contributed to the restricted development of the embryos even death.

### Abnormal Formation of Labyrinth Layer in *CreER* ^*T2*^ */SynA* ^*−/−*^ Placenta

**FIGURE 2:**
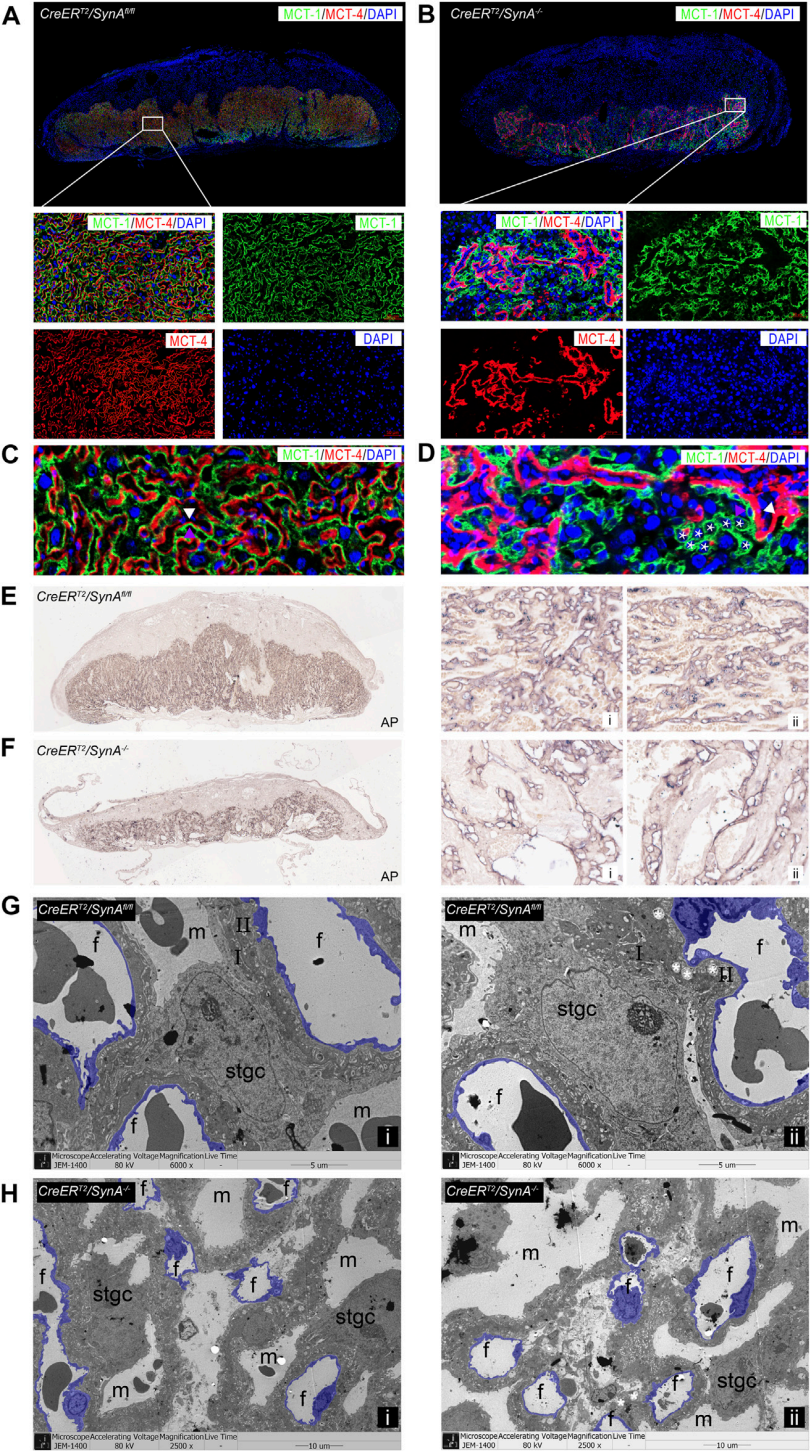
Abnormal formation of the labyrinth layer in the *CreER* ^*T2*^ */SynA* ^*−/−*^ placenta. **(A,B)** Anti-MCT1 and anti-MCT4 immunofluorescence staining of the *CreER* ^*T2*^ */SynA* ^*fl/fl*^ and *CreER* ^*T2*^ */SynA* ^*−/−*^ placentas at E17.5 d. Anti-MCT1 and anti-MCT4 are expressed in the labyrinth, which showed a reduced labyrinth area in the *CreER* ^*T2*^ */SynA* ^*−/−*^ placenta. **(C,D)** Unfused trophoblast cells gathered in the labyrinth layer, surrounding the morphologically abnormal STB-II, indicating that STB-I was disrupted. Moreover, the STB-II was reduced after Syncytin-A disruption. **(E,F)** AP staining of *CreER* ^*T2*^ */SynA* ^*fl/fl*^ and *CreER* ^*T2*^ */SynA* ^*−/−*^ placentas at E17.5 d. Overall figure (4×), partial figure (20×). **(G,H)** Transmission electron microscopy observation of endothelial cells (purple) in placental tissues at E17.5 d. **(G)** (6,000×), **(H)** (2,500×, for better insight on vascularization in *CreER* ^*T2*^ */SynA* ^*−/−*^ placentas). m: maternal lacuna; f: fetal vessel; I: STB-I; II: STB-II; *: lipid inclusion.

The labyrinth is composed of two syncytiotrophoblast layers and Stgcs that separate the maternal lacunae and fetal blood vessels responsible for maternal-fetal exchange. To further characterize the placental defects in the labyrinth, STB-I and STB-II were immunofluorescently labeled with anti-MCT1 and anti-MCT4 antibodies. Detailed histological analysis identified abnormal formation of the labyrinth in *CreER* ^*T2*^ */SynA* ^*−/−*^ placentas. A decreased labyrinth layer was observed and large number of unfused trophoblast cells in STB-I accumulated in the labyrinth layer, surrounding the morphologically abnormal STB-II, indicating STB-I disruption (Figures 2A,B). Interestingly, STB-II was reduced after Syncytin-A disruption, suggesting that Syncytin-A may also be involved in the formation of STB-II (Figures 2C,D). Stgcs marked by AP staining showed a similar distribution with STB-I in the labyrinth, indicating that Stgcs were also affected in Syncytin-A disruption placenta. Also, the maternal blood sinuses were remarkably irregular, consistent with the changes of STB-I and STB-II (Figures 2E,F). The ultrastructure observation of placenta tissues showed smaller and irregular vessels in *CreER* ^*T2*^ */SynA* ^*−/−*^ placenta compared to regular distribution vessels in *CreER* ^*T2*^ */SynA* ^*fl/fl*^ placenta, which indicating a disruption of placental vascularization and syncytiotrophoblast layers after *syncytin-a* knockout (Figures 2G,H, Supplementary Figure S1).

### Cell Apoptosis and Vascular Disruption in *CreER* ^*T2*^ */SynA* ^*−/−*^ Placenta

**FIGURE 3:**
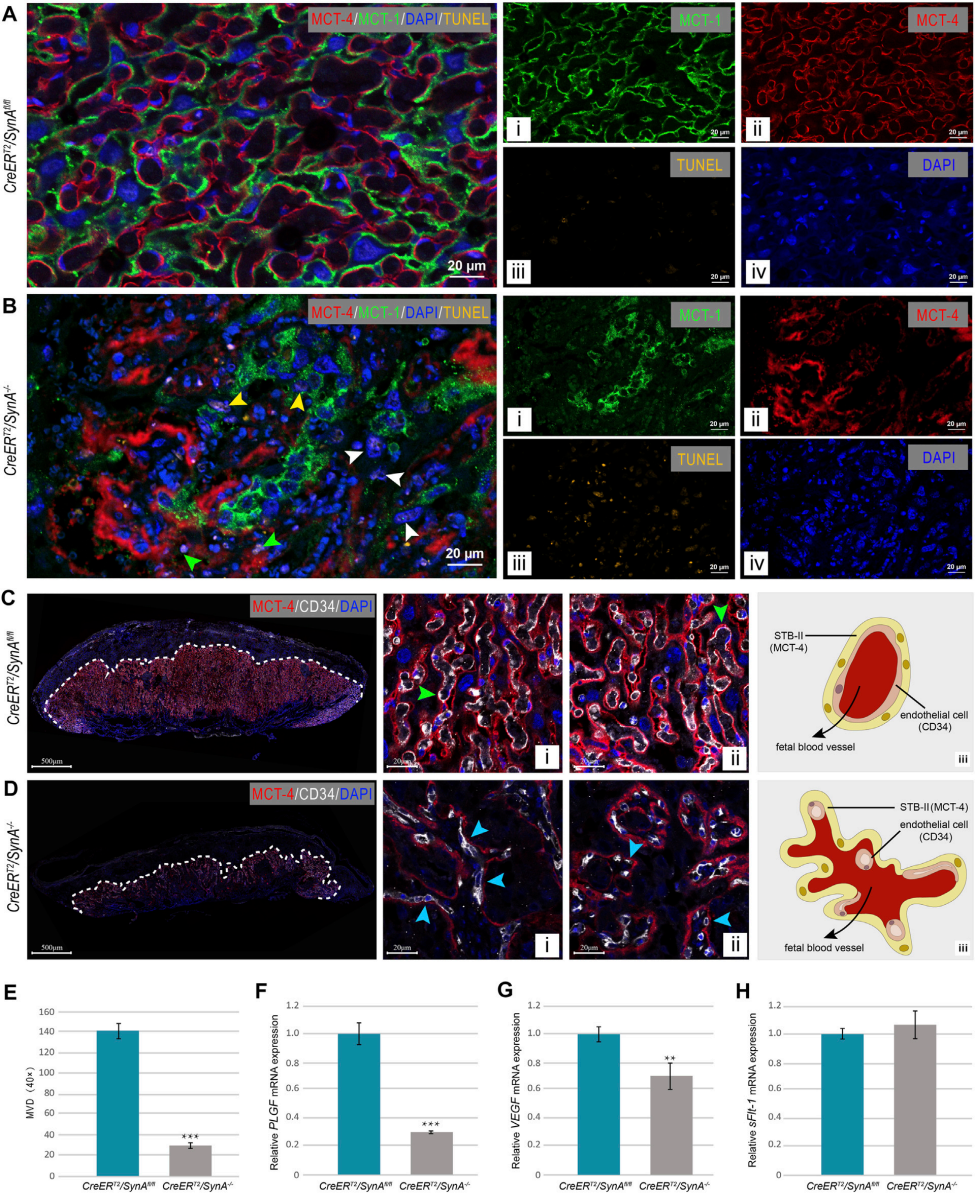
***CreER*** ^***T2***^ ***/SynA*** ^***−/*** *−*^ placenta exhibit cell apoptosis and vascular deficiency. **(A,B)** TUNEL staining of placental tissues at E 17.5 d. TUNEL positive cells were observed in *CreER* ^*T2*^ */SynA* ^*−/−*^ placentas compared to *CreER* ^*T2*^ */SynA* ^*fl/fl*^ placenta. **(C,D)** Anti-MCT4 and CD34 immunofluorescence staining of *CreER* ^*T2*^ */SynA* ^*fl/fl*^ and *CreER* ^*T2*^ */SynA* ^*−/−*^ placentas (E17.5 d). Fetal blood vessels are displayed with STB-II fully and tightly (green arrowheads) in the *CreER* ^*T2*^ */SynA* ^*fl/fl*^ placenta [**(C)**-i, -ii], but were lost in the normal structure and scattered with only a small surface attached to STB-II (blue arrowheads) in *CreER* ^*T2*^ */SynA* ^*−/−*^ placenta [**(D)**-i, -ii]. Distribution schematics of the endothelial cells are shown in **(C)**-iii and **(D)**-iii, and suggest a disruption of placental vascularization after disruption of Syncytin-A. **(E)** MVD of *CreER* ^*T2*^ */SynA* ^*−/−*^ placenta was decreased (E17.5 d). ***: *p* < 0.001. **(F–H)** Relative mRNA expression levels of PLGF, VEGF and sFlt-1 of *CreER* ^*T2*^ */SynA* ^*fl/fl*^ and *CreER* ^*T2*^ */SynA* ^*−/−*^ placentas (E17.5 d). **: *p* < 0.01, ***: *p* < 0.001.

Compared with *SynA* ^*fl/fl*^ placentas, apoptotic cells were found in *SynA* ^*−/−*^ placenta. Co-staining of MCT-1 and MCT-4 showed that apoptotic cells were unfused STB-I (yellow arrow), STB-II (green arrow) and Stgc (white arrow) (Figures 3A,B). To further determine the vascularization of placenta, endothelial cells were stained for CD34 by immunofluorescence. Fetal blood vessels were regularly distributed and surrounded by STB-II in the *SynA* ^*fl/fl*^ placenta, while decreased and deformed vessels were observed in the *SynA* ^*−/−*^ placenta (Figures 3C,D). The average MVD of *SynA* ^*−/−*^ placentas was decreased compared with *SynA* ^*fl/fl*^ placentas, when observed from E14.5 to E18.5 d, indicating that placental angiogenesis was impaired after Syncytin-A disruption (Figure 3E). Moreover, the morphology of fetal vessels was changed. Normally, fetal blood vessels are closely attached to STB-II and present as moderately dilated lumens (Figures 3C-i, -ii). However, compressed and flattened vessels were observed in *SynA* ^*−/−*^ placenta (Figures 3D-i, -ii). Notably, tube formation was also impaired along with a reduction in the number of vessels (Figures 3C-iii, 3D-iii). Consistent with morphological changes, the mRNA expression of VEGF and PLGF were decreased in *SynA* ^*−/−*^ placentas, in the absence of any change in mRNA expression of sFlt-1 (Figures 3F,G). Thus, changes in angiogenic factors may contribute to the abnormal placental vascularization after the disruption of Syncytin-A.

### Impaired Tube Formation of HUVECs Co-Cultured With Syncytin-1 Disruption Cell Medium

**FIGURE 4:**
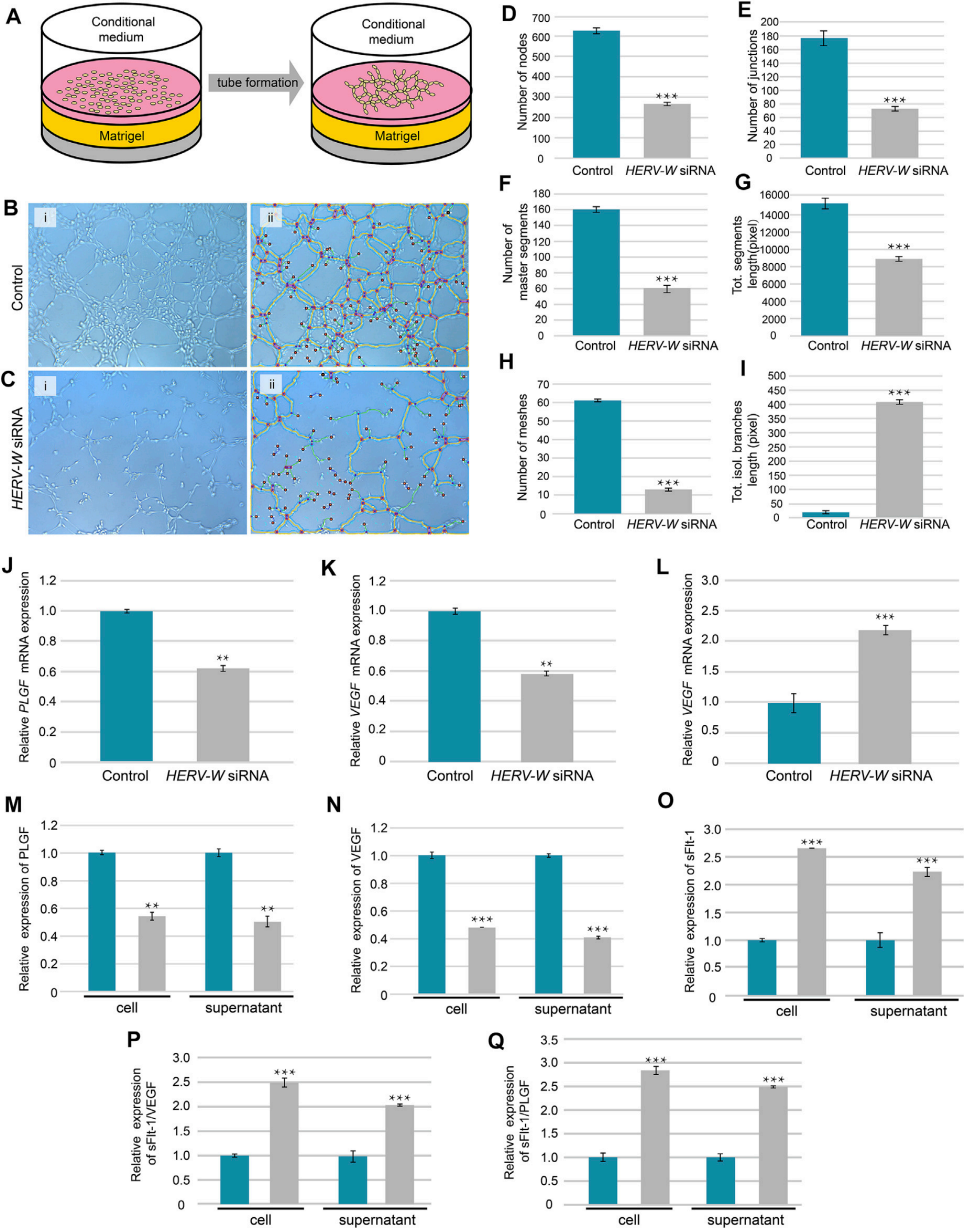
Induced tube formation of HUVECs by conditioned medium from Syncytin-1-knockdown and control trophoblasts. **(A)** Schematic of tube formation by using HUVECs co-cultured with conditional supernatant from trophoblast cells. **(B,C)** Tube formation pictures were taken at 2 h after cultured with conditional medium from control and *HERV-W* siRNA-transfection cells. **(D–I)** Parameters for assessing the ability of tube formation. **(J–O)** Relative mRNA and protein expression levels of PLGF, VEGF and sFlt-1 in cells and cellular supernatant from control and *HERV-W* siRNA-transfected cells. **(P,Q)** Relative protein expression of sFlt-1/VEGF and sFlt-1/PLGF. **: *p* < 0.01, ***: *p* < 0.001.

Tube formation is essential for placental development, and failed tube formation was observed in the *SynA* ^*−/−*^ placenta (Figure 3B-I and -ii). To further explore the relationship between syncytin and angiogenesis, we tested the ability of trophoblast-conditioned medium, from Syncytin-1 (*HERV-W*)-silenced trophoblast cells, to induce tube formation by HUVECs *in vitro* (Figure 4A). The results showed a decreased ability to induce HUVEC tube formation by conditional medium from both BeWo and HTR-8/SVneo cells after knock down of Syncytin-1 (Figures 4B–I). Moreover, the mRNA and protein expression levels of VEGF and PLGF, in both cells and media, were both decreased after Syncytin-1 knockdown, resulting in a decrease in ability of trophoblast-conditioned medium to induce HUVEC tube formation (Figures 4J–O). Also, the ratios of sFlt-1/VEGF and sFlt-1/PLGF were high, providing additional evidence for abnormal angiogenesis (Figures 4P,Q).

### Syncytin-1 Disruption Suppressed Angiogenesis *via* the PI3K/Akt/mTOR Signaling Pathway in Trophoblast Cells

**FIGURE 5:**
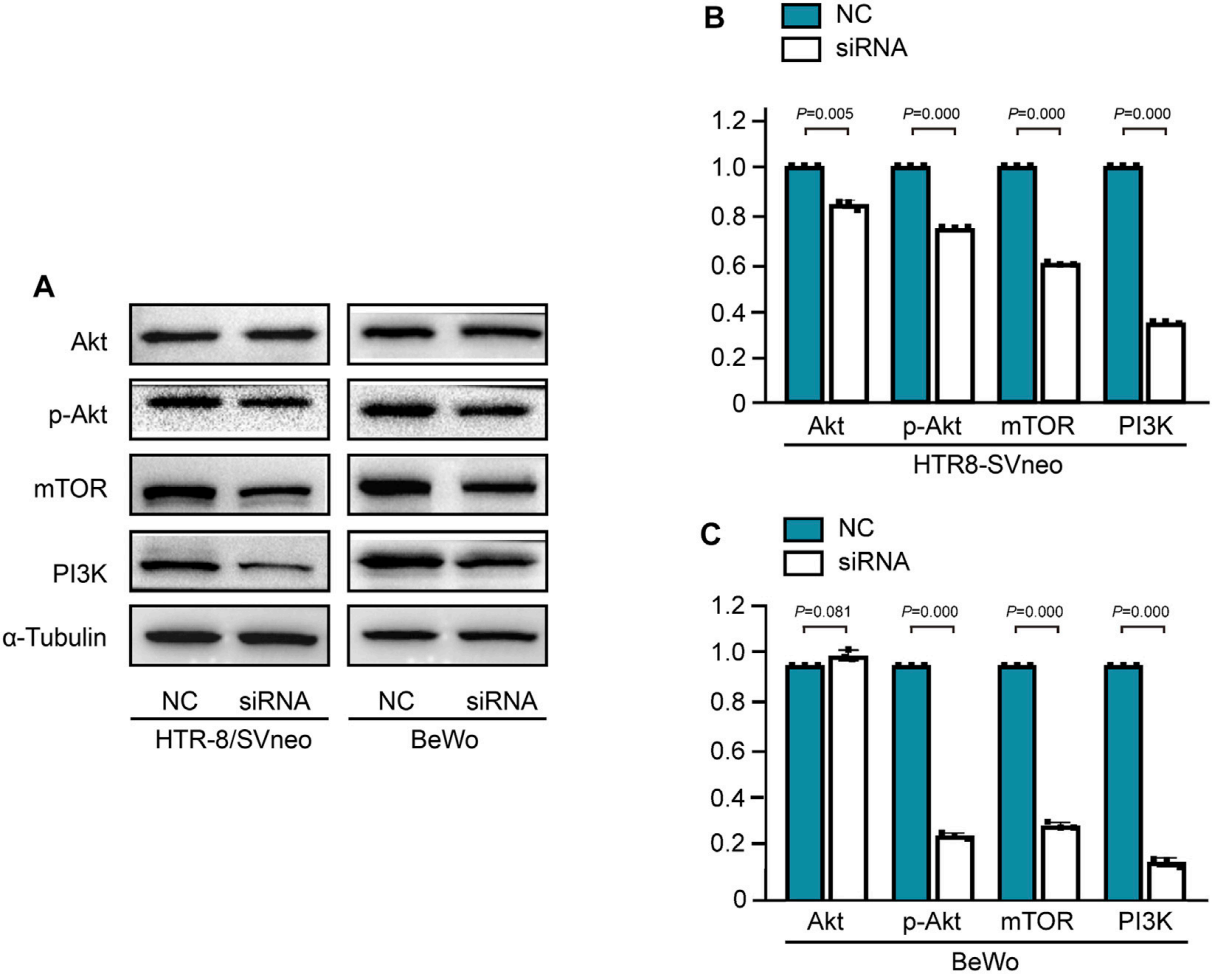
Syncytin-1 disruption suppressed angiogenesis via the PI3K/Akt/mTOR signaling pathway in trophoblast cells **(A)**. Western blotting analysis of PI3K, P-Akt and mTOR proteins in BeWo and HTR-8/SVneo trophoblast cells after Syncytin-1 disruption, with *α*-Tubulin used as loading control **(B, C)**. The expression levels of PI3K, P-Akt and mTOR were significantly decreased in *HERV-W*-silenced trophoblast cells compared with control group. ***: *p* < 0.001.

The PI3K/Akt pathway is essential for angiogenesis during the process of embryogenesis (Karar and Maity, 2011). To further determine the potential pathway, PI3K/Akt/mTOR pathway was detected in *HERV-W*-silenced trophoblast cells. The protein expression levels of PI3K, P-Akt and mTOR were decreased in BeWo and HTR-8/SVneo trophoblast cells after Syncytin-1 disruption, which related with low expression of VEGF (Figure 5).

## Discussion

Syncytin is known to play a critical role in placental development. Fusogenic and non-fusogenic functions of syncytin have been explored in placental pathogenesis, which may result in PE, FGR and abortion (Huang et al., 2013; Huang et al., 2014). Before our research, Anne Dupressoir et al. found that homozygous *syncytin-a* null mouse embryos die between E11.5 and E13.5 d *in utero*, which suggesting an unsuccessful and unsustainable gestation in mice (Dupressoir et al., 2009). Such a knockout mice model could not well explore the role of syncytin in the placenta during the whole gestation. Considering the importance of full gestation for the exploration of syncytin function, we generated the inducible knockout of *syncytin-a* mouse model to knockout *syncytin-a* gene in both spatial and temporal dimensions (Qiao et al., 2017). As an endogenous retroviral envelop protein, syncytin is specifically expressed in placental trophoblast cells (Gong et al., 2005). Cre-ER^T2^ recombinase activity can be induced by tamoxifen to knockout *syncytin-a* gene in placental specifically (Imai et al., 2001).

Here, we characterized placenta formation in the *CreER* ^*T2*^ */SynA* ^*fl/fl*^ mouse model following knockout of *syncytin-a* gene by administering tamoxifen on E11.5 d. The results showed that the disruption of *syncytin-a* gene leads to impaired placental development, especially abnormal formation of the labyrinth layer, the unfused STB-I and irregular formation of STB-II. Also, STBs and Stgc were identified as apoptotic cells, indicating that apoptosis was involved in defects after *syncytin-a* disruption, as previous research suggested (Dupressoir et al., 2011; Huang et al., 2014). Furthermore, abnormal placental vascularization was observed from E14.5 to E18.5 d, indicating that placental angiogenesis is impaired after Syncytin-A disruption, which could be explained by an imbalance of angiogenic and antiangiogenic factors, specifically decreased PLGF and VEGF. What’s more, the spaces that endothelial cell inhabit were completely abnormal in morphology and structure, as the STB-II showed. This may also be another mainly cause of disrupted vascularization. Disrupted spaces and poor vascularization created a vicious cycle. The resultant irregular formation of the maternal-fetal exchange structure with *syncytin-a* deficiency may cause the absorption of embryos *in utero*. The altered development and growth of blood vessels were impaired due to a strongly abnormal morphogenesis of STB-I and STB-II after *syncytin-a* disruption.

Angiogenic factors are likely to be important in the regulation of placental angiogenesis (Wheeler et al., 1995; Clark and Charnock-Jones, 1998; Chen and Zheng, 2014). The low expression of VEGF, PLGF and overexpression sFlt-1 are observed in PE and may lead to placental ischemia (Zárate et al., 2014; Schrey-Petersen and Stepan, 2017; Bhattacharjee et al., 2021). Here we noticed that disruption of Syncytin-A resulted in abnormal placental angiogenesis. However, the low expression of Syncytin-A may not be causative, but rather reflective of poor trophoblast function due to placental ischemia. Therefore, the function of trophoblast cells might be of more interest in the pathogenesis of PE or other placental-related diseases. As a key protein, the exploration of syncytin functions is very important for understanding the functions of trophoblast cells. Here, we first identified the regulation of syncytin on placental angiogenesis both *in vivo* and *in vitro*, although this function relies on the disruption of trophoblast cells. Cell culture studies have demonstrated that syncytin can impair the expression of proangiogenic factors and diminish tube formation of HUVECs *in vitro*. And the abnormal angiogenesis is dependent on suppressed PI3K/Akt/mTOR signaling pathway after syncytin disruption.

In contrast to the previous view that the formation of STB-II is induced by Syncytin-B independently (Dupressoir et al., 2005; Dupressoir et al., 2011), our experiments show irregular structure formation of STB-II after the disruption of Syncytin-A, suggesting a new role of Syncytin-A in the fusogenic activities of STB-I and STB-II, although the mechanism remains unknown. TMEM16F was reported to play an essential role in placental trophoblast fusion by translocating phosphatidylserine to the cell surface, independent of apoptosis, to participate in cell-cell fusion (Zhang et al., 2020). The relationship between TMEM16F and syncytin in the fusogenic activity of trophoblast cells is still unclear, but TMEM16F has been shown to specifically impair STB-II, not STB-I, suggesting a potential relationship between TMEM16F and Syncytin-A in the formation of STB-II.

**FIGURE 6:**
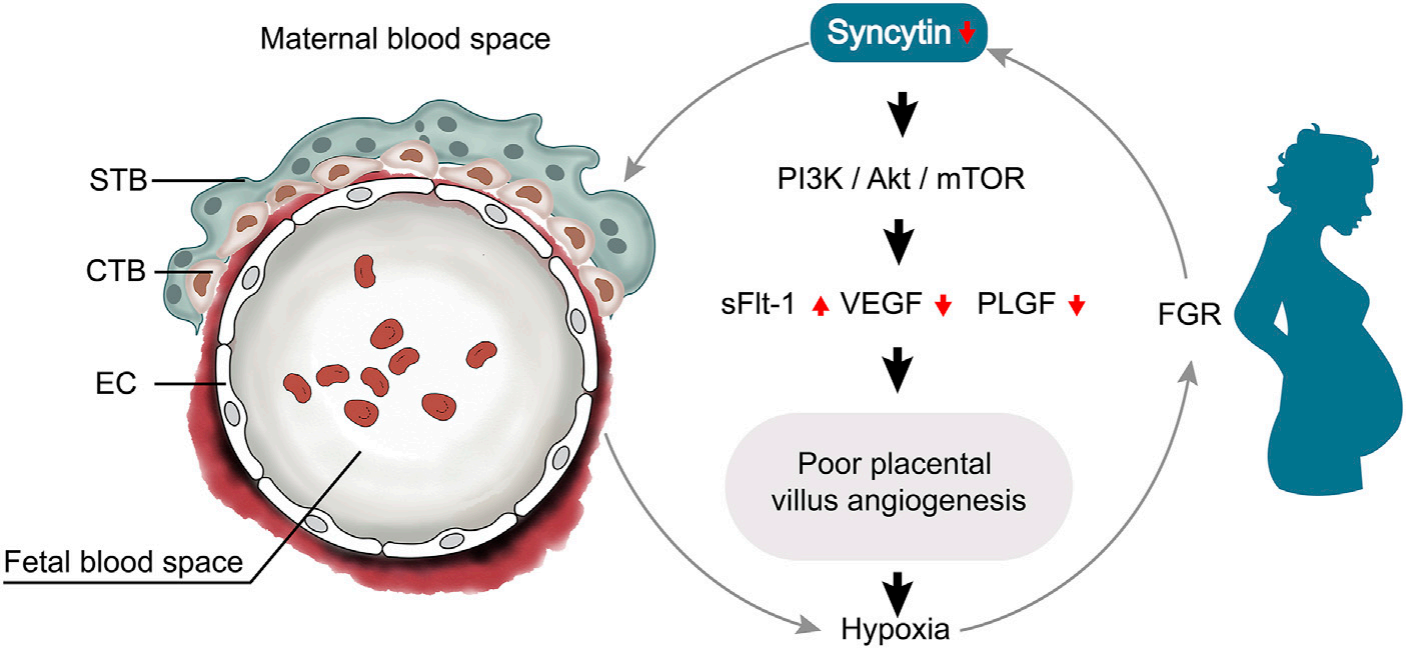
Relationship network diagram among syncytin and placental angiogenesis. Syncytin disruption suppressed angiogenesis through deactivated PI3K/Akt/mTOR signaling pathway with low expression of VEGF and PLGF, and the poor angiogenesis led to placental hypoxia which causing a vicious cycle.

Syncytin is known as fusogenic protein involved in the process of trophoblast formation. With more understanding of syncytin and its role in placental pathology, it is apparent that low expression of syncytin is associated with altered trophoblast cells, resulting impaired placental angiogenesis. Syncytin disruption suppressed angiogenesis through deactivated PI3K/Akt/mTOR signaling pathway with low expression of VEGF and PLGF, and the poor angiogenesis led to placental hypoxia which causing a vicious cycle (Figure 6). However, it is unclear what the direct relationships are between Syncytin and endothelial cells. Our current finding shows that Syncytin-A disruption specifically impairs formation of the two syncytiotrophoblast layers and fetal blood vessels, but further studies are required to dissect the relationship between syncytin and endothelial cells.

## Conclusion

In this study, the formation of two syncytiotrophoblast layers and fetal blood vessels is disrupted due to Syncytin-A deficiency in mice. Consistent with the defects observed *in vivo*, medium conditioned by trophoblast cells, following Syncytin-1 knockdown, contains lower levels of VEGF and PLGF, but higher levels of sFlt-1, and is reduced in ability to induce HUVEC tube formation by suppressed the PI3K/Akt/mTOR signaling pathway. To conclude, we give a new sight on understanding syncytin and pathophysiology of placenta-related disease.

## Data Availability

The original contributions presented in the study are included in the article/Supplementary Material, further inquiries can be directed to the corresponding author.

## Supplementary Material

Supplementary Figure 1 Transmission electron microscopy observation of STB-I (green), STB-II (yellow), endothelial cells (purple) and Stgc (pink) in placental tissues at E17.5 d. m: maternal lacuna; f: fetal vessel.
