# Supplementary figures and images for: The Role of Syncytin in Placental Angiogenesis and Fetal Growth
Source: Front Cell Dev Biol. 2022 Apr 12;10:852561. doi: 10.3389/fcell.2022.852561 (PMC9039138; doi:10.3389/fcell.2022.852561)

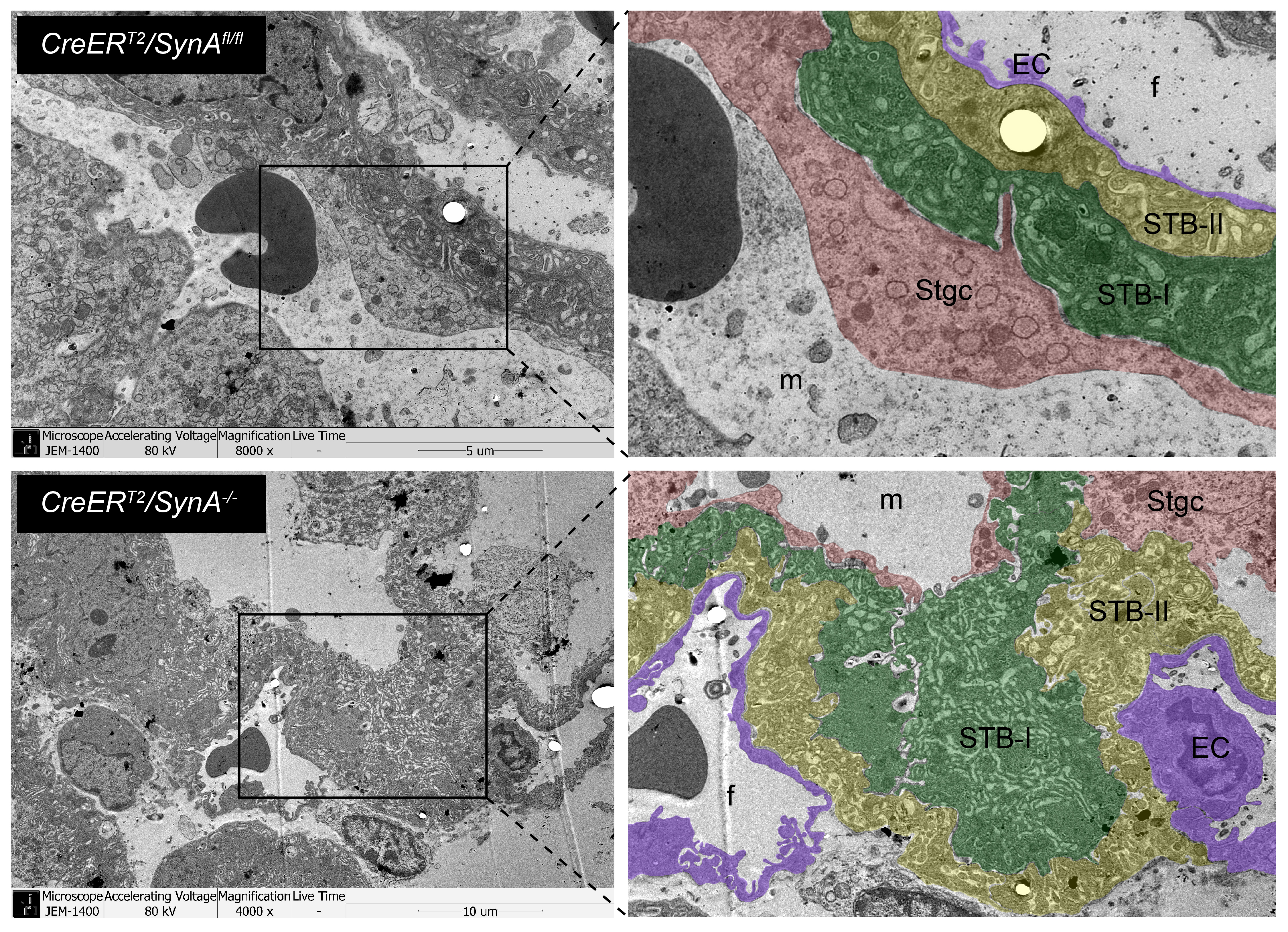

Supplement: Supplementary file 1 [file Image1.jpeg]
